# Caustic ingestion in adults: The role of endoscopic classification in predicting outcome

**DOI:** 10.1186/1471-230X-8-31

**Published:** 2008-07-25

**Authors:** Hao-Tsai Cheng, Chi-Liang Cheng, Cheng-Hui Lin, Jui-Hsiang Tang, Yin-Yi Chu, Nai-Jen Liu, Pang-Chi Chen

**Affiliations:** 1 Division of Digestive Therapeutic Endoscopy, Chang Gung Memorial Hospital, Tao-Yuan, Taiwan; 2 Chang Gung University College of Medicine, Tao-Yuan, Taiwan

## Abstract

**Background:**

The ingestion of caustic substances induces an extensive spectrum of injuries to the aerodigestive tract which include extensive necrosis and perforation of the esophagus and stomach. The gold standard of safely assessing depth, extent of injury, and appropriate therapeutic regimen is esophagogastroduodenoscopy (EGD). The objective of this study was to report our clinical experience and to evaluate the role of a 6-point EGD classification system of injury in predicting outcomes in adult patients diagnosed with caustic agent ingestion.

**Methods:**

The study was a retrospective medical chart review from 273 patients admitted to the Chang Gung Memorial Hospital in Tao-Yuan, Taiwan between June 1999 and July 2006 for treatment of caustic ingestion. The patients underwent EGD within 24 hours of admission and mucosal damage was graded using Zagar's modified endoscopic classification scheme. After treatment, patients were followed in the outpatient clinic for a minimum of 6 months.

**Results:**

A total of 273 patients were included for analysis. Grade 3b injury was the most common caustic injury (n = 82, 30.03%), followed by grade 2b injuries (n = 62, 22.71%). Stricture was the most common complication (n = 66, 24.18%), followed by aspiration pneumonia (n = 31, 11.36%), and respiratory failure (n = 21, 7.69%). Compared to grade 3a mucosal injury, grade 3b mucosal injuries were at greater risk of prolonged hospital stay (odds ratio [OR]: 2.44; 95% confidence interval [CI]: 1.25–4.80), ICU admission (OR: 10.82; 95% CI: 2.05–200.39), and gastrointestinal (OR: 4.15; 95% CI: 1.55–13.29) and systemic complications (OR: 4.07; 95% CI: 1.81–14.07).

**Conclusion:**

In patients with caustic ingestion, EGD should be performed within 12 to 24 hours and categorized according to a 6-point scale. Patients with grade 3b burns identified on endoscopy have high rates of morbidity. The 6-point scale is useful for predicting immediate and long-term complications, and guiding appropriate therapy.

## Background

The ingestion of caustic substances induces a wide range of injuries to the gastrointestinal tract, which can be mild or fatal, or lead to chronic disease [1]. Caustic ingestion in children is usually accidental ingestion [2], while ingestion by adults is often due to suicidal intent, and injuries tend to be more severe [3].

Caustic agents with a pH level <2 or >12 rapidly penetrate layers of the esophagus resulting in necrosis-induced eschar formation in the mucosa that limits deep tissue penetration [4]. The extent of tissue destruction depends on the physical form, type, and concentration of corrosive agent, premorbid state of the tissue, contact duration, and amount of substance ingested. Esophageal mucosa is thought to be more resistant to acidic than alkaline substances, as alkaline liquids are often highly viscous and thus persist for a longer duration in the esophageal mucosa [5]. Liquefaction necrosis occurs and serious esophageal injury becomes inevitable once alkaline liquids penetrate deep muscle layers [6].

The gold standard of safely assessing depth, extent of caustic ingestion injury, and appropriate therapeutic regimen is esophagogastroduodenoscopy (EGD). Indications, mucosal injury classification, optimal timing, and the degree of esophageal injuries that necessitate EGD in relation to treatment regimens, however, are matters of debate [4-10]. The objective of this study was to report our clinical experience and to evaluate the role of a 6-point EGD classification system of injury in predicting outcomes and guiding therapy in adult patients diagnosed with caustic agent ingestion.

## Methods

A retrospective chart review of 288 adult patients (>18 years of age) who were admitted to Chang Gung Memorial Hospital, Tao-Yuan, Taiwan, for caustic ingestion between June 1999 and July 2006 was conducted. Parameters analysed were age, gender, intent of ingestion, substance ingested and amount, time to expiration, ICU admittance, length of hospital stay, complications, and the severity of mucosal injury as assessed by EGD.

EGD with a standard upper GI endoscope was performed by experienced physicians within 24 hours of ingestion. Endoscopes used were Olympus GIF XQ-230, GIF Q-240X, and GIF Q-260, with diameters of 9.2 mm, 9.4 mm, and 9.2 mm, respectively (Olympus, Tokyo, Japan). Oral cavity xylocaine spray was used for anaesthesia except in 15 cases, which received ventilation support under general anaesthesia because of respiratory difficulty (n = 11) or unclear consciousness (n = 4). Gentle insufflations and retrovisual methods were performed carefully or avoided in the presence of severe stomach injury. Mucosal damage was graded using a modified endoscopic classification described by Zagar et al [11] (Table 1).

**Table 1:** Zargar's grading classification of mucosal injury caused by ingestion of caustic substances

Grade 0	Normal examination
Grade 1	edema and hypermia of the mucosa
Grade 2a	Superficial ulceration, erosions, friability, blisters, exudates, hemorrhages, whitish membranes
Grade 2b	Grade 2a plus deep discrete or circumferential ulcerations
Grade 3a	Small scattered areas of multiple ulceration and areas of necrosis with brown-black or greyish discoloration
Grade 3b	Extensive necrosis

Patients were treated with a proton pump inhibitor or H2 antagonist and were maintained without oral intake until their condition was considered stable. Patients received parenteral nutrition during this period. If infection was suspected, antibiotics (a 1^st ^generation cepholasporin and gentamicin) were administered after blood cultures were obtained. If a patient's condition destablized or respiratory difficulty was encountered, they were transferred to the intensive care unit for further evaluation. After discharge, patients were followed in the outpatient clinic for at least 6 months. Any complications observed during follow-up were recorded. Upper GI complications included bleeding, perforation, and stricture formation. Bleeding was defined as melena, hematemasis, and/or coffee-ground vomitus. Perforation was diagnosed by the presence of free air on a plain chest radiograph. Stricture was defined as dysphagia, symptoms of regurgitation, or difficulty in swallowing with confirmation by endoscopy, esophagogram, and/or upper GI radiography. Systemic complications included renal insufficiency, liver damage, diffuse intravascular coagulation, and hemolysis. Liver damage was defined as an elevation in the serum level of alanine aminotransferase or asparatate aminotransferase greater than 3 times the upper normal limit. Renal insufficiency was defined as a plasma creatinine level of >1.4 mg/dL in the absence of other renal diseases. Criteria for disseminated intravascular coagulation and/or hemolysis were prolonged plasma coagulation time, decreased fibrinogen or antithrombin levels, and decreased platelet count.

Demographic data were described by mean and standard deviations for normally distributed continuous variables, median and interquartile range for non-normally distributed continuous variables, and frequencies and percentages for categorical variables. Wald's Chi-Square tests adjusted for age obtained by generalized estimation equations were used to evaluate for overall survival and complications over grade of mucosal injury. Data subset was subsequently analyzed using logistic regression. Data were analyzed using SAS 9.0 (SAS Institute Inc, Cary, NC, US), and *P *< 0.05 was considered significant.

## Results

A total of 273 patients consisting of 127 (47%) males and 146 females (53%) with a mean age of 43.77 ± 18.46 were included in our analysis (Table 2). One patient attempted suicide twice with different corrosive substance in a 3-month period. Fifteen patients were excluded from analysis as a result of missing data (n = 14) or endoscopy failure due to severe laryngeal edema (n = 1). Ingestion intent was primarily attributed to suicide (n = 194, 71.06%) while 28.94% (n = 79) of the cases were accidental. The amount of ingested substance ranged from 2 ml to 3000 ml and was estimated based on the history given by the patient or family member. Industrial cleaning agents containing lye or other alkaline chemical (ie, caustic soda, drain cleaners, machine cleaners, and deacidification products containing sodium hydroxide or sodium-potassium hydroxide, dishwater detergents) or caustic acids were considered caustic substances. Ingestion of industrial cleaning agents (n = 131, 47.99%) and strong acids (n = 95, 34.80%) comprised the majority of cases. Other caustic substances such as pesticides, caustic food, drugs, and other substances comprised the rest of the cases. Of these, 35.16%, 34.43%, and 30.40% were alkaline, hydrochloric acid, or unidentified acid-based substances, respectively.

**Table 2:** Patient demographic features and caustic ingestion characteristics by endoscopic grade of mucosal injury

**Variables**	**Overall**	**Endoscopic Grade**						
	**(n = 273)**							
		
		**0**	**1**	**2a**	**2b**	**3a**	**3b**	***P***
		**(n = 3)**	**(n = 31)**	**(n = 56)**	**(n = 62)**	**(n = 39)**	**(n = 82)**	
Age	43.77 ± 18.46	37.00 ± 27.78	40.45 ± 15.54	42.21 ± 18.96	40.92 ± 16.72	44.10 ± 17.78	48.32 ± 19.94	0.0107*
Male	127 (46.52)	3 (100)	16 (5.93)	31 (11.48)	26 (9.63)	16 (5.93)	35 (12.96)	0.1534
Accidental Ingestion	79 (28.94)	1 (33.33)	10 (32.26)	19 (33.93)	14 (22.58)	15 (38.46)	20 (24.39)	0.3820
Class of Substance								
Alkaline	96 (35.16)	3 (100)	9 (29.03)	22 (39.29)	24 (38.71)	13 (33.33)	25 (30.49)	0.5302
Acid								
HCL	94 (34.43)	0 (0.00)	12 (38.71)	13 (23.21)	23 (37.10)	11 (28.21)	35 (42.68)	0.1498
Other Acid	83 (30.40)	0 (0.00)	10 (32.26)	21 (37.50)	15 (24.19)	15 (38.46)	22 (26.83)	0.4086
								
Type of Substance								
Industrial cleaning	131 (47.99)	2 (66.67)	15 (48.39)	26 (46.43)	28 (45.16)	18 (46.15)	42 (51.22)	0.9574
Drug	3 (1.10)	0 (0.00)	0 (0.00)	2 (3.57)	1 (1.61)	0 (0.00)	0 (0.00)	0.2862
Pesticide	13 (4.76)	0 (0.00)	1 (3.23)	5 (8.93)	3 (4.84)	4 (10.26)	0 (0.00)	0.0194*
Strong acid	95 (34.80)	1 (33.33)	11 (35.48)	15 (26.79)	24 (38.71)	14 (35.90)	30 (36.59)	0.7028
Food	12 (4.40)	0 (0.00)	2 (6.45)	2 (3.57)	2 (3.23)	2 (5.13)	4 (4.88)	0.9265
Others	19 (6.96)	0 (0.00)	2 (6.45)	6 (10.71)	4 (6.45)	1 (2.56)	6(7.32)	0.7013
Package Volume (ml)	100 (2–3000)	20 (10–100)	50 (30–100)	100 (35–150)	100 (50–150)	100 (50–120)	100 (50–200)	0.4423

* *P *< 0.05 statistical significance

Data presented as median (range) or N (%), endoscopic grade 0 is not included in the comparison for small contribution and possible confounding

The results of EGD in this study showed that grade 3b injuries were the most common caustic injury (n = 82, 30.04%), followed by grade 2b injuries (n = 62, 22.71%) (Table 2). Of the 82 grade 3b patients, the esophagus was inspected in 100% of patients, the stomach was inspected in 98% (80/82), and duodenum was inspected in 84% (69/82). Severe injuries were observed in the stomach (n = 116, 42.50%), the duodenum (n = 120, 43.1%), and the esophagus (n = 71, 26.00%). Age distribution among grades of mucosal injury was significantly different (*P *= 0.0107, Table 2) and was subsequently used as an adjusting factor.

Table 3 illustrates select variables that influenced caustic injury survival and associated complications compared with the grades of mucosal injury. Overall, the mean hospital stay was 8 days (range 0–90); hospital mortality was 6.59% (18/273); and 29 patients were admitted to the ICU. Deaths occurred from 3 days to 2 months after ingestion of the substance as a result of esophageal perforation (n = 1), tracheal perforation with active bleeding (n = 1), hematemesis with sudden apnea (n = 4), lung cancer (n = 1), or multiple organ failure (n = 11). Seventy-six patients (27.8%) developed GI complications and 20.5% (56/273) of patients developed systematic complications. Stricture formation was the most common complication observed in all patients (n = 66, 24.18%) and patients with grade 3b mucosal injury (n = 44, 53.66%), followed by aspiration pneumonia (n = 31, 11.36% vs. n = 20, 24.39%) and respiratory failure (n = 21, 7.69% vs. n = 16, 19.51%).

**Table 3:** Select parameters compared with endoscopic grade of mucosal injury

**Variables**	**Overall** ** (n = 273)**	**Endoscopic Grade**						
		
		**0** ** (n = 3)**	**1 ** **(n = 31)**	**2a ** **(n = 56)**	**2b ** **(n = 62)**	**3a ** **(n = 39)**	**3b ** **(n = 82)**	***P***^†^
Hospital Stay (days)	8 (0, 90)	4 (3, 10)	2 (1, 51)	4.5 (0, 87)	7 (2, 44)	9 (0, 33)	13 (0, 90)	0.0147*
Expired	18 (6.59)	0 (0.0)	0 (0.0)	1 (1.79)	2 (3.23)	1 (2.56)	14 (17.07)	0.0648
Time to Expired (days)	15.5 (3, 56)	NA	NA	11 (11, 11)	16 (15, 32)	16 (16, 16)	15 (3, 56)	--
ICU Admission	29 (10.62)	0 (0.0)	2 (6.45)	2 (3.57)	5 (8.06)	1 (2.56)	19 (23.17)	0.0244*
Systemic Complication	56 (20.51)	1 (33.33)	3 (9.68)	4 (7.14)	11 (17.74)	5 (12.82)	32 (39.02)	0.0111*
Aspiration Pneumonia	31 (11.36)	1 (33.33)	2 (6.45)	1 (1.79)	4 (6.45)	3 (7.69)	20 (24.39)	0.0068*
Respiratory Failure	21 (7.69)	0 (0.0)	2 (6.45)	1 (1.79)	2 (3.23)	0 (0.0)	16 (19.51)	0.0023*
DIC	10 (3.67)	0 (0.0)	0 (0.0)	1 (1.79)	1 (1.61)	0 (0.0)	8 (9.76)	0.1524
Hepatic	10 (3.67)	0 (0.0)	0 (0.0)	1 (1.79)	4 (6.45)	2 (5.13)	3 (3.66)	0.6841
Renal	7 (2.56)	0 (0.0)	0 (0.0)	1 (1.79)	1 (1.61)	0 (0.0)	5 (6.10)	0.4300
Gastrointestinal Complication	76 (27.84)	0 (0.0)	0 (0.0)	4 (7.14)	9 (14.52)	12 (30.77)	51 (62.20)	<0.0001*
Stricture	66 (24.18)	0 (0.0)	0 (0.0)	2 (3.57)	9 (14.52)	11 (28.21)	44 (53.66)	<0.0001*
Bleeding	13 (4.76)	0 (0.0)	0 (0.0)	2 (3.57)	1 (1.61)	2 (5.13)	8 (9.76)	0.3656
Perforation	6 (2.20)	0 (0.0)	0 (0.0)	1 (1.79)	0 (0.0)	0 (0.0)	5 (6.10)	0.2306
Fistula	2 (0.73)	0 (0.0)	0 (0.0)	1 (1.79)	0 (0.0)	0 (0.0)	1 (1.22)	0.6302

^†^Wald's Chi-Square test

*P < 0.05 statistical significance

Data presented as median (range) or number (%). Endoscopic grade 0 is not included in the comparison secondary to small contribution.

Stricture formation typically occurred 2 weeks after caustic ingestion. Management of the 66 patients with a stricture included gastrojejunostomy (n = 24), dilation with endoscope (n = 21), medical treatment (n = 10), esophagectomy (n = 5), jejunostomy (n = 4), esophago-colonic bypass (n = 1), and nasogastric feeding due to old CVA (n = 1). Of the 21 patients dilated endoscopically, 11 patients required subsequent surgery due to perforation (n = 3, one in the esophagus, two in the pyloric area) and failure of dilation (n = 8). Gastrojejunostomy were performed due to gastric outlet obstruction or EC junction stricture. The time of operation was determined by the patient's symptoms and signs. Fifty-one patients received surgery due to perforation (n = 6) and stricture (n = 34), and 11 patients required surgery after endoscopic dilation. Four deaths (in 51 patients who required surgery) were due to multiple organ failure, sepsis, or hematemesis.

The majority of complications were observed in patients with grade 3b burns, and these were more likely to result in prolonged hospital stay (n = 13), death (n = 14), and ICU admission (n = 19). Statistical significance was observed in duration of hospital stay, ICU admittance, systematic complications, aspiratory pneumonia, respiratory failure, GI complications, and GI stricture among patients with different grades of mucosal injury (all *P *< 0.05; Table 3).

Table 4 shows the odds ratio of endoscopic grades 2a versus 2b, and 3a versus 3b among selected variables. Grade 2b mucosal injuries were 2.5 times more likely to result in longer hospital stay (95% CI: 1.32–4.87, *P *< 0.05) than 2a. Other variables analysed did not show a statistically significant difference. Grade 3b mucosal injuries were 2.4 times more likely to result in longer hospital stay (95% CI: 1.25–4.80, *P *< 0.05), and 10.8 times more likely to be admitted to the ICU (95% CI: 2.05–200.39, *P *< 0.05), than grade 3a injuries. Additionally, patients with grade 3b burn injuries were 4.1 times more likely to develop systematic complications (95% CI: 1.55–13.29, *P *< 0.05), 4.07 times more likely to develop GI complications (95% CI: 1.81–9.69, *P *< 0.05) and 3.34 times more likely to develop stricture (95% CI: 1.47–8.09, *P *< 0.05) than those with grade 3a burns.

**Table 4:** Select parameters and odds ratio of endoscopic grade 2a versus 2b, 3a versus 3b

**Variables**	**Endoscopic Grading ** **2a vs. 2b ** **OR (95% CI)**	***P***	**Endoscopic Grading ** **3a vs.3b ** **OR (95% CI)**	***P***
Median Hospital Stay (days)	2.52 (1.32, 4.87)	0.0052*	2.44 (1.25, 4.80)	0.0102*
Expired (days)	1.88 (0.17, 41.13)	0.6124	7.17 (1.32, 133.49)	0.0640
ICU Admission	2.37 (0.49, 17.02)	0.3159	10.82 (2.05, 200.39)	0.0241*
Systemic Complication				
Overall	2.89 (0.92, 11.03)	0.0871	4.15 (1.55, 13.29)	0.0083*
Aspiratory Pneumonia	4.12 (0.57, 83.15)	0.2163	3.58 (1.10, 16.22)	0.0553
Respiratory Failure	1.77 (0.16, 38.89)	0.6453	--	--
DIC	0.91 (0.04, 23.27)	0.9443	--	--
Hepatic	3.74 (0.53, 74.44)	0.2448	0.82 (0.13, 6.48)	0.8280
Renal	0.95 (0.04, 24.54)	0.9710	--	--
Gastrointestinal Complication				
Overall	2.10 (0.66, 8.47)	0.2183	4.07 (1.81, 9.69)	0.0010*
Stricture	4.56 (1.11, 30.86)	0.0596	3.34 (1.47, 8.09)	0.0053*
Bleeding	0.41 (0.02, 4.50)	0.4794	2.05 (0.48, 14.07)	0.3818
Perforation	--	--	--	--
Fistula	--	--	--	--

* *P *< 0.05 indicates statistical significance.

Table 5 shows select variables compared with acid and alkali ingestion. Statistical significance was observed in duration of hospital stay only (*P *= 0.0419).

**Table 5:** Select parameters compared with alkali and acid ingestion groups

**Variables**	**Alkali** ** (n = 96)**	**Acid**		***P***^†^
			
		**HCL ** **(n = 94)**	**Other acid ** **(n = 83)**	
Hospital Stay (days)	8 (4,16)	8 (3,14)	7 (3,13)	0.0419*
Expired	6 (6.25)	18 (6.59)	7(8.43)	0.8073
Time to Expired (days)	15 (11–16)	15.5 (11–23)	22 (13–32)	--
ICU Admission	12 (12.50)	29 (10.62)	8 (9.64)	0.5074
Systemic Complication	27 (28.13)	56 (20.51)	13 (15.66)	0.0525
Aspiration Pneumonia	13 (13.54)	31 (11.36)	8 (9.64)	0.5188
Respiratory Failure	10 (10.42)	21 (7.69)	6 (7.23)	0.1874
DIC	4 (4.17)	10 (3.66)	4 (4.82)	0.4754
Hepatic	7 (7.29)	10 (3.66)	1 (1.20)	0.0511
Renal	3 (3.13)	7 (2.56)	2 (2.41)	0.6618
GI Complication	36 (37.50)	76 (27.84)	15 (18.07)	0.0818
Stricture	31 (32.29)	66 (27.84)	12 (14.46)	0.1855
Bleeding	7 (7.29)	13 (4.76)	3 (3.61)	0.1776
Perforation	4 (4.17)	6 (2.20)	2 (2.41)	0.0728
Fistula	1 (1.04)	2 (0.73)	1 (1.20)	0.4492

^†^Wald's Chi-Square test

* *P *< 0.05 statistical significance

Data presented as median (range) or number (%).

## Discussion

The results of this study confirm Zargar's endoscopic classification of mucosal injuries post caustic ingestion in relation to clinical outcome. Grade 3b mucosal injury assessed by EGD was a predictor of prolonged duration of hospital stay, ICU admittance, and GI and systematic complications. Over 80% of patients with grade 3 burns develop stricture formation, while one-third of those with grade 2 develop pyloric stenosis, acid regurgitation, and perforation [11-13]. In our data, only 50% of patients with grade 3 burns developed stricture formation, while 10% of those with grade 2 developed GI complication. Our lower results may be because of the development and use of more effective anti-acid medications (proton pump inhibitors, H2 antagonists) and more aggressive use of nasogastric irrigation to reduced effect of the substance ingested [6]. The primary reason for ingestion in our patient population was suicidal intent (71%); thus, the injury produced was generally greater and more extensive than that in individuals who ingest caustic substances out of curiosity or by accident [14].

Caustic ingestion typically refers to the ingestion of strongly alkaline or acidic household or industrial cleaning products. Alkalis can be found in drain openers, bleaches, toilet bowl cleaners, and detergents containing hydrogen peroxide or sodium hydroxide at concentrations from 4% to 54% [5]. Solid alkaline variants such as crystals or particles adhere to the mucous membrane and increase esophageal injury as a result of prolonged contact with the mucosa [5]. Acid ingestion, which tends to occur less frequently in Western countries (<5%), is more common in countries like Taiwan where hydrochloric acid and sulphuric acid (found in toilet bowl cleaners, antirust compounds, battery fluids, and commercial pesticides) are readily accessible [7].

Earlier studies have questioned the recommendation of routine endoscopic evaluation of all patients after presumed caustic ingestion [15,16] on the basis that in the absence of symptoms following unintentional ingestion severe injury is unlikely. The tensile strength of healing tissues in the first 3 weeks is low due to an absence of collagen. New collagen formation does not begin until the second week after injury. Thus, it is advocated that endoscopy should be avoided from 5 to 15 days after caustic ingestion [11]. Currently, EGD evaluation within 12 hours and no later than 24 hours after caustic ingestion is considered safe, and may be beneficial up to 96 hours after ingestion [17,18]. EGD is not recommended from 2 to 3 days up to 2 weeks after caustic ingestion as a result of wound softening.

Early classification of caustic substance induced injuries may be beneficial in predicting outcomes [19,20]. Though there are no strict guidelines regarding when endoscopy is indicated, ingestion of larger amounts of corrosives, persistent symptoms, as well as suicidal intention are considered indications for endoscopy in the absence of a third degree burn of the hypopharynx [21,22]. Flexible endoscopy and concurrent endoscopic ultrasound using a high-frequency catheter probe have decreased the rate of perforation that occurs with rigid instruments [8]. This study suggests that patients with mucosal damage exceeding grade 2a are at a higher risk of developing serious complications, while patients with mild mucosal damage have a significantly reduced mortality and morbidity. Death in our patients with grade 2a injury (n = 1) was due to tracheoesophageal fistula, sepsis and acute bleeding; with 2b injuries (n = 2) was lung cancer and sudden apnea, and with 3a injury (n = 1) was hematemesis with sudden apnea. In the patients with grade 2b and 3a injuries, ICU observation and nutritional support may be mandatory if there are any signs of bleeding and the patient experiences abdominal pain, and antibiotics are cautiously recommended in those with lung involvement. Patients with grade 3a lesions may not require immediate surgery [11,23,24].

## Conclusion

In conclusion, the results of this study indicate that patients with findings of grade 3b burns on endoscopy have high the risk of perforation and complications. Endoscopy done within 12 hours and no later than 24 hours following caustic ingestion to classify mucosal injury subsequent to caustic ingestion is useful to determine the severity of injury, particularly in suicidal cases, and thus helpful in predicting outcomes. A 6-point grading system of mucosal injury, rather than a 4- or 5-point system is useful for predicting immediate and long-term complications, and guiding appropriate therapy.

## Competing interests

The authors declare that they have no competing interests.

## Authors' contributions

C-LC and C-HL carried out the molecular genetic studies, participated in the sequence alignment and drafted the manuscript. J-HT`and Y-YC carried out the immunoassays. P-CC participated in the sequence alignment. N-JL participated in the design of the study and performed the statistical analysis. H-TC conceived of the study, and participated in its design and coordination and helped to draft the manuscript. All authors read and approved the final manuscript.

## Pre-publication history

The pre-publication history for this paper can be accessed here:


